# Characteristics of Ambient Black Carbon Mass and Size-Resolved Particle Number Concentrations during Corn Straw Open-Field Burning Episode Observations at a Rural Site in Southern Taiwan

**DOI:** 10.3390/ijerph13070688

**Published:** 2016-07-08

**Authors:** Yu-Hsiang Cheng, Li-Sing Yang

**Affiliations:** Department of Safety, Health and Environmental Engineering, Ming Chi University of Technology, 84 Gungjuan Rd., Taishan, New Taipei 24301, Taiwan; lisingyang@gmail.com

**Keywords:** black carbon, particle number, corn straw, open-field burning

## Abstract

Information on the effect of open-field burning of agricultural residues on ambient black carbon (BC) mass and size-resolved particle number concentrations is scarce. In this study, to understand the effect of such open-field burning on short-term air quality, real-time variations of the BC mass and size-resolved particle number concentrations were monitored before and during a corn straw open-field burning episode at a rural site. Correlations between the BC mass and size-resolved particle number concentrations during the episode were investigated. Moreover, the particle number size distribution and absorption Ångström exponent were determined for obtaining the characteristics of aerosol emissions from the corn straw open-field burning. The results can be used to address public health concerns and as a reference for managing similar episodes of open-field burning of agricultural residues.

## 1. Introduction

Open-field biomass burning includes a large variety of combustion activities, including postharvest burning of agricultural fields, residential wood burning, and wildfires, and it is a major source of particulate matter (PM), carbon monoxide, methane, and volatile organic compounds in the atmosphere. Moreover, it has a substantial impact on global climate change and adverse effects on human health [1,2,3,4,5,6,7].

A large amount of PM is produced during uncontrolled burning of agricultural residues, immediately degrading the air quality in the surrounding areas and causing acute symptoms in people with chronic respiratory conditions [3,5,8]. The dispersion of smoke can also cause freeway traffic accidents by drastically reducing visibility. Hence, in Taiwan, open-field burning of agricultural residues is prohibited by the Environmental Protection Administration. Nevertheless, for farmers, open-field burning of agricultural residues is an inexpensive method of preparing a field for the next crop, and it can remove residues, rapidly release nutrients, and increase agricultural production in the next growing cycle. 

Thus, open-field burning of agricultural residues continues in rural areas of Taiwan despite the prohibition policy. Although the economic and practical benefits of burning agricultural residues are apparent, recognizing the environmental burden of and health risks associated with this activity is imperative.

Epidemiological studies have consistently reported that short-term exposure to high concentrations of PM can increase cardiovascular morbidity and mortality [9,10,11]. Therefore, short-term air pollution episodes resulting from open-field burning of agricultural residues could pose a serious public health hazard. Ryu et al. [8] noted that, in rural areas of Korea, the average concentrations of PM_2.5_ and PM_2.5__–10_ (particles with aerodynamic diameters in the range 2.5–10 μm) were 67.9 and 18.7 μg·m^−3^ during barley straw burning periods, respectively; these parameter values were 35.6 and 13.3 μg·m^−3^ during normal periods, respectively. A substantial increase in the concentration of PM_2.5_ was observed during a barley straw burning period, and the value reached 110.3 μg·m^−3^; moreover, the PM_2.5_/PM_10_ (particles with aerodynamic diameters less than 10 μm) ratio was 0.79. Cheng et al. [12] reported average PM_2.5_ and PM_2.5__–10_ concentrations as high as 123.6 and 31.5 μg·m^−3^ during rice straw burning periods in Taichung, Taiwan, respectively; by contrast, the concentrations were 32.6 and 21.4 μg·m^−3^ for non-waste burning periods, respectively. Furthermore, the main ionic species in the fine aerosol produced by the burning were Cl^–^, K^+^, and NO_3_^–^. Differences in the observed PM_2.5_ concentrations during different agricultural residue burning periods could result from differences in the combustion conditions, meteorological conditions, and straw type. The PM emission factor for open-field burning of agricultural residues has been estimated in numerous studies, and the estimates differ considerably from one another [13,14,15,16]. Nevertheless, the PM_2.5_ levels could increase by up to a few times during agricultural residue burning relative to the levels during normal periods, indicating the potentially acute risk posed to human health. 

Particularly, toxicological studies have suggested that ultrafine particles (UFPs; particle size less than 100 nm) may be more harmful to health than large particles are because small particles have a considerably larger adsorbed or condensed surface area than large particles with the same mass do and can be deposited in the alveoli, where they interact with epithelial cells [17,18,19]. Additionally, black carbon (BC) is a major constituent of atmospheric aerosols, and it has an appreciable impact on global climate change, apart from adversely affecting human health [7,20,21,22]. Bond et al. [7] reported that global emissions of BC in 2000 were as high as 7.6 Tg, with approximately 40% of the BC originating from open-field biomass burning. 

Considerable information on the magnitude of BC mass and particle number concentrations in urban environments is available [23,24,25,26]. In urban air, over 80% of airborne particles are UFPs [27,28]. UFPs in urban areas originate from traffic emissions, and their concentrations are in the range of 1.0 × 10^4^ to 2.0 × 10^4^ cm^−3^ [29,30,31,32,33,34]. Additionally, in urban environments, BC aerosols are typically emitted from transportation. Generally, the BC mass concentrations in urban areas are approximately 1000–7500 ng·m^−3^, and the fraction of BC in the PM_2.5_ mass concentration is approximately 10%–15% [23,24,25,26,33,34,35,36,37]. In urban areas, the time variation of the BC mass concentration is similar to that of the particle number concentration, clearly indicating that most BC and UFPs are likely released from the same emission source; furthermore, the BC mass concentration is positively correlated with particle number concentrations, particularly with the number concentrations of particles in the size range of 56–180 nm [25,34,38,39]. 

However, information on the effect of open-field burning of agricultural residues on ambient BC mass and size-resolved particle number concentrations is scarce. In this study, to understand the effect of open-field burning on short-term air quality, real-time variations of the BC mass and size-resolved particle number concentrations were monitored before and during a corn straw burning episode at a rural site. Correlations between the BC mass and size-resolved particle number concentrations during the episode were investigated. Moreover, the particle number size distribution and absorption Ångström exponent were determined for obtaining the characteristics of aerosol emissions from the corn straw open-field burning. The results can be used to address public health concerns and as a reference for managing similar episodes of open-field burning of agricultural residues. 

## 2. Methods 

### 2.1. Monitoring Site, Monitoring Equipment, and Data Collection 

In 2014, the harvested areas of feed and food corn in Taiwan were 13,544 and 13,461 ha, respectively, and the production of these crops was 6.22 × 10^7^ and 1.04 × 10^8^ kg, respectively [40]. Furthermore, approximately 54% and 15% of feed and food corn were produced in the Tainan area in Southern Taiwan, respectively. In this area, every year, the main harvest periods are from January to February for feed corn and November to December for food corn. Therefore, ambient BC mass and size-resolved particle number concentrations were monitored in the postharvest period from 11 to 19 February 2014 in the Tainan area. 

**Figure 1:**
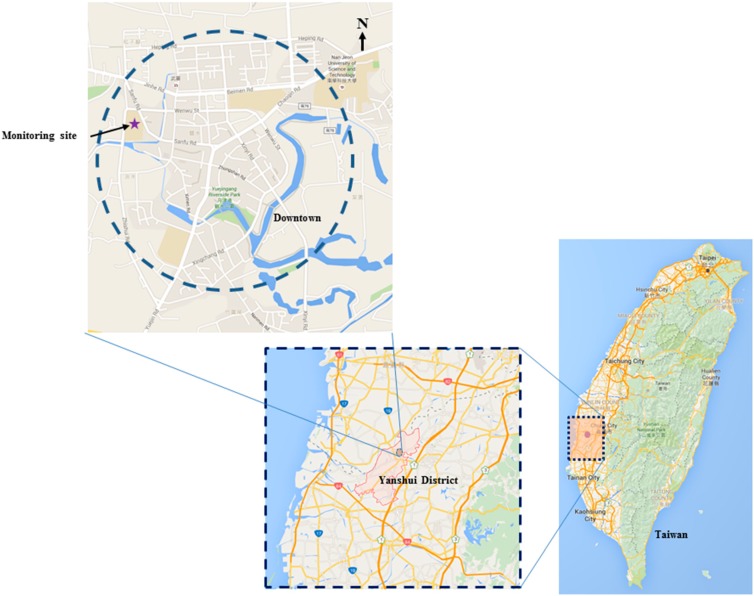
Monitoring location in this study.

Figure 1 shows the monitoring location in this study. The monitoring location was in the downtown area of Yanshui. There are a significant number of corn fields in the vicinity of this small town. In this study, the monitoring instruments were set up on the rooftop of a building of Yanshui Junior High School (23°19′17.5″N, 120°15′50.3″E). This school is located at the west edge of the downtown area and adjacent to corn fields in the north and west. An aethalometer (Model AE31; Magee Scientific Corp., Berkeley, CA, USA) was used to measure the BC mass concentrations at a sampling flow rate of 2 L·min^−1^. This instrument can be used to evaluate BC mass concentrations at seven wavelengths: 370, 470, 520, 590, 660, 880, and 950 nm. Additionally, a scanning mobility particle sizer (SMPS) spectrometer (TSI Model 3938; TSI, Inc., Shoreview, MN, USA) was employed to measure particle number size distributions of particles with diameters in the range of 13–750 nm. These two instruments were operated continuously from 11 to 19 February 2014, and the logging interval for all measurements was set at 5 min. Local meteorological data were recorded using a Vantage Pro2^TM^ weather station (Davis Instruments Corp., Hayward, CA, USA). 

During the whole monitoring period, a firework display was performed near this monitoring site on 14 February 2014. The effect of the firework display on ambient BC mass and particle number concentrations had been reported by Lin et al. [41] and it would not be addressed as an issue in this study. Despite the firework display on 14 February 2014, a few events of agricultural residue burnings were clearly observed from 15 to 17 February 2014. A remarkable open-field burning of corn straw upwind of the monitoring location was observed between 17:00 and 19:00 (2 h) on 16 February 2014. The extremely high BC mass and particle number concentrations came from the open-field burning located approximately 400 m north of the monitoring site. Based on the meteorological data and the distance between the burning and monitoring sites, the plume age was approximately 4–7 min when it was detected in this study. During this period, the ambient air was filled with smoke. Therefore, the data obtained during this period were considered to correspond to a short-term air pollution episode for agricultural residue burning (episode period). Furthermore, the data monitored at the same time and for the same duration on 12 February 2014, were considered to represent the reference air quality status (non-episode period) for this monitoring site because the wind speed and principal wind direction on this day were similar to those on 16 February 2014. The strength of the correlation between the BC mass and the particle number during the episode and non-episode periods was determined using the Pearson product-moment correlation coefficient (*R_Pearson_*). The significance level was 0.05 for all statistical tests.

### 2.2. Absorption Ångström Exponent Estimation 

Jeong et al. [42] noted that BC concentrations measured at a wavelength of 370 nm could be considerably higher than those measured at a wavelength of 880 nm during a forest fire episode because of a marked increase in ultraviolet-absorbing organic compounds. This finding points to the abundance of organic compounds in the particles released into the atmosphere during a forest fire. In the current study, the BC mass concentrations determined at the wavelengths of 370 and 880 nm were treated as ultraviolet BC (UVBC) and BC, respectively, to distinguish particles bound to ultraviolet-absorbing organic compounds [26,37,43,44]. Furthermore, BC mass concentrations evaluated at the wavelengths of 470 and 950 nm were used to determine the absorption Ångström exponent, which can be used as an index to identify the types of BC emission sources [44,45,46,47].

The absorption Ångström exponent α can be computed from the aerosol light absorption at two different wavelengths in the ultraviolet to infrared spectral region. In this study the aerosol light absorption coefficients at 470 and 950 nm wavelengths were used:

(1) $$\alpha =\frac{\text{ln}\left(b_{\text{abs,λ}=470}/b_{\text{abs,λ}=950}\right)}{\text{ln}\left(\text{950/470}\right)}$$

where *b*_abs, λ = 470_ and *b*_abs, λ = 950_ are the absorption coefficients of the aerosol at 470 and 950 nm wavelengths, respectively. The absorption coefficient of the aerosol can be determined from the change rate of ATN:

(2) $$b_{\text{abs}}=\frac{\text{dATN}}{dt}\cdot \frac{A}{Q}\cdot \frac{1}{C}$$

where *C* is a light enhancement parameter, it is associated with multiple scattering of the light beam at the filter fibers in the unloaded filter, and it is strongly dependent on the filter material [48]. In this study, *C* was set as 2.14 for the quartz filter [48]. The ATN values were measured from the aethalometer. 

## 3. Results and Discussion 

### 3.1. Meteorological Conditions during the Reference and Event Days

**Figure 2:**
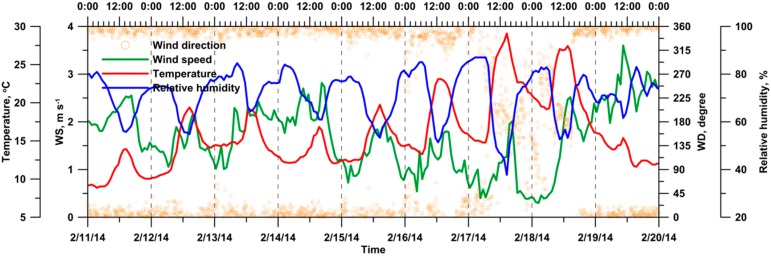
Time variations of meteorological conditions at the monitoring site during the whole monitoring period.

During the whole monitoring period, the hourly average wind speed, temperature, and relative humidity were 1.7 m·s^−1^, 15.9 °C, and 71.4%, respectively (Figure 2). Despite the irregular wind direction patterns on 17 and 18 February 2014, the principal wind direction was NNW–NNE. 

**Figure 3:**
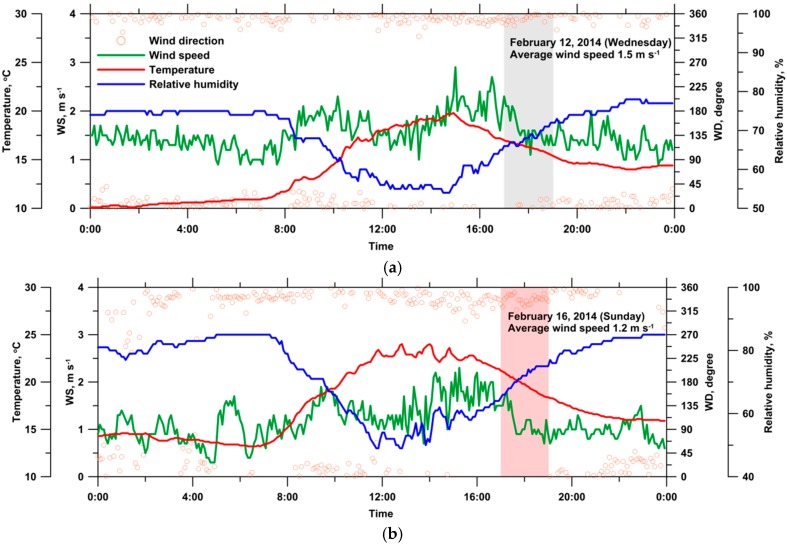
Diurnal variations of meteorological conditions at the monitoring site on (**a**) the reference day; and (**b**) the event day.

Figure 3 presents the diurnal variation of the 5-min meteorological data at the monitoring site on the reference (12 February 2014) and event (16 February 2014) days. The daily average wind speed, principal wind direction, temperature, and relative humidity were 1.5 m·s^−1^, NNW–NNE, 14.3 °C, and 68.9%, respectively, on the reference day. On the event day, the daily average wind speed, principal wind direction, temperature, and relative humidity were 1.2 m·s^−1^, NW–NE, 17.8 °C, and 73.2%, respectively. 

Table 1 summarizes the meteorological data on the reference day at the time and for the duration identical to those of the episode. During open-field burning of corn straw (episode duration), the 5-min wind speed was between 0.9 and 1.8 m·s^−1^ (1.1 m·s^−1^ on average), and the principal wind direction was between NW and N.

### 3.2. BC Concentration and Absorption Ångström Exponent Value during the Reference and Event Days

**Figure 4:**
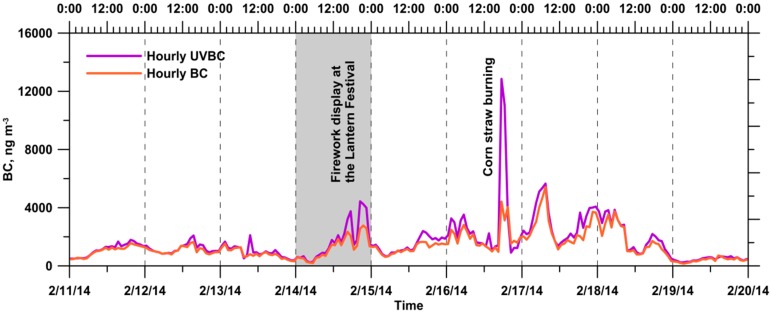
Time variations of the hourly UVBC and BC concentrations at the monitoring site during the whole monitoring period.

The time variations of hourly UVBC and BC concentrations at the monitoring site during the whole monitoring period were presented in Figure 4. During the whole monitoring period, the hourly average UVBC and BC mass concentrations were 1652 and 1345 ng·m^−3^, respectively. The interquartile ranges were 799–1921 and 681–1641 ng·m^−3^ for hourly UVBC and BC mass concentrations, respectively. Despite the firework display on 14 February 2014 and a remarkable corn straw burning on 16 February 2014, the highest daily average BC mass concentration was observed on 17 February 2014, and it was 2540 ng·m^−3^. This highest daily average BC mass concentration was due to some sporadic agricultural residue burnings at the different upwind sites of the monitoring location. 

However, the short-term effect of the open-field burning on ambient BC mass concentration on 17 February 2014 was not more serious than that on 16 February 2014. The probable reason was that the levels of measured BC could be influenced by the relative locations and distances between those burning sites and the monitoring location. Moreover, the ambient BC mass concentrations could also be influenced by the local meteorological conditions (Figure 2). The irregular wind direction pattern was recorded on 17 February 2014. Furthermore, the lowest daily average BC mass concentration was observed on 19 February 2014 due to its highest daily average wind speed and it was only 411 ng·m^−3^. According to the long-term measurement results of Cheng et al. [26], the BC mass concentration was 3330 ng·m^−3^ in the Kaohsiung urban area in Southern Taiwan. Despite the open-field burning, the BC mass concentration in the rural area considered in the current study was lower than that in the urban area. Boogaard et al. [24] also found that BC mass concentrations on major streets in five Dutch cities were 2100–4400 ng·m^−3^, approximately 1.5–2.2 times higher than those at urban background locations.

**Figure 5:**
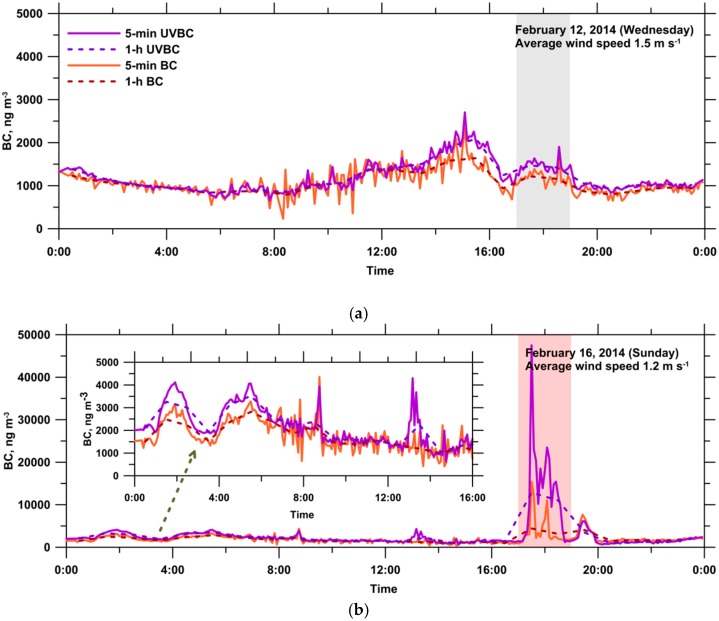
Diurnal variations of 5-min and 1-h UVBC and BC concentrations at the monitoring site on (**a**) the reference day; and (**b**) the event day.

Figure 5 shows the diurnal variations of the 5-min and 1-h UVBC and BC concentrations at the monitoring site on the reference and event days. The temporal variation of the 5-min UVBC and BC concentrations during daytime was considerable, and it was associated with a change in the wind direction. The daily average BC mass concentrations on the reference and event days were 1074 and 2013 ng·m^−3^, respectively. The daily average BC mass concentration on the event day was significantly higher than that on the reference day because obvious open-field burning was performed. On the reference day, the lowest BC concentration, 860 ng·m^−3^, was observed between 04:00 and 09:00. Subsequently, the BC concentrations increased steadily from 09:00 to 15:00. The highest BC concentration occurred between 14:00 and 16:00, and the value was approximately twice the lowest value. The BC concentrations decreased gradually from 15:00 to 00:00 and returned to the lowest level. Between 14:00 and 22:00, the UVBC concentrations were considerably higher than the BC concentrations, indicating the presence of BC aerosols bound to organic compounds, which enhanced UV absorption. Similar results were found on other days during the monitoring period. The positive Delta-C (UVBC–BC) values likely corresponded to an increase in household activities and residential wood burning emissions after midday, and they were observed until 22:00. Therefore, anthropogenic combustion activities were the major source of BC aerosols during this period in the rural area. Similar results have been reported by Husain et al. [49] and Wang et al. [37]. Wang et al. [37] suggested that the gradual buildup of Delta-C values likely corresponded to an increase of residential wood during emissions after sunset. On the event day, an abrupt increase in the UVBC and BC concentrations was clearly observed between 17:00 and 19:00, particularly in UVBC values. Past results have indicated a relatively large amount of polycyclic aromatic hydrocarbons (PAHs) in aerosols produced from agricultural residue burning [5,13,50,51,52,53]. Yang et al. [51] demonstrated that rice straw burning was a major source of PAHs. The ambient concentrations of PAHs in particulate and gaseous phases were 33 and 1160 ng·m^−3^, respectively, on the days when rice straw was burnt. These values were greater than the concentrations on non-burning days. Cheng et al. [12] reported that the average organic carbon (OC) and elemental carbon (EC) concentrations were as high as 35.1 and 8.9 μg·m^−3^ during rice straw burning periods, respectively, whereas they were 4.0 and 0.7 μg·m^−3^ during non-burning periods, respectively. These previous findings suggest the presence of BC aerosols bound to organic compounds during the episode. The organic compounds on BC aerosols could enhance UV absorption.

The absorption Ångström exponent has been used as an index for determining the type of BC emission source. Kirchstetter et al. [45] found that the absorption Ångström exponent value ranged between 0.8 and 1.1 for diesel soot, and Sandradewi et al. [44] observed that the absorption Ångström exponent values for vehicular traffic and wood burning were 1.1 and 1.9, respectively. Furthermore, Lewis et al. [46] reported that the absorption Ångström exponent value for rice straw burning was as high as 2.8. Favez et al. [47] noted that the absorption Ångström exponent value for agricultural waste burning organic aerosols was approximately 3.5. Notably, the values of the absorption Ångström exponent presented in previous studies were determined from two different wavelengths in the ultraviolet-to-infrared spectral region, which may explain the different values that have been obtained in various studies. A high absorption Ångström exponent value was obtained from the wood combustion aerosol because the aerosol was bound to compounds showing strong absorption in the ultraviolet region. In this study, the daily average absorption Ångström exponent value for the reference day was 1.2, which was same as the value on the event day. The BC concentrations at this rural monitoring site on normal days were lower, and the measurement results indicated that vehicular traffic was the main source of BC aerosols. Nevertheless, the hourly absorption Ångström exponent value during the episode was 2.0, implying that the source of BC aerosols during the episode was related to agricultural residue burning. 

### 3.3. Total Particle and Ultrafine Particle Number Concentrations during the Reference and Event Days 

**Figure 6:**
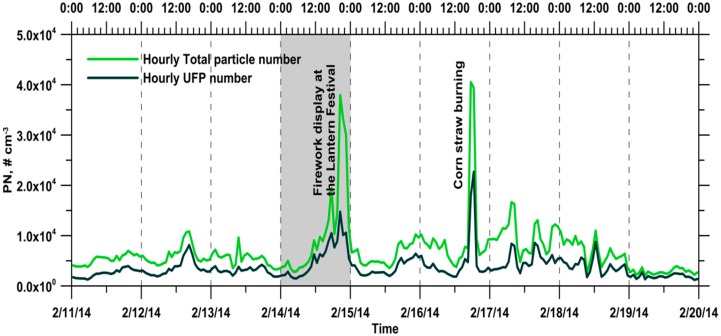
Time variations of the hourly total particle and UFP number concentrations at the monitoring site during the whole monitoring period.

In this study, the total particle and UFP number concentrations in the size ranges of 13–750 and 13–100 nm were calculated from the raw data of the SMPS, respectively. During the whole monitoring period, the hourly average total particle and UFP number concentrations were 7.06 × 10^3^ and 3.76 × 10^3^ cm^−3^, respectively. The interquartile ranges were 4.18 × 10^3^–8.13 × 10^3^ and 2.22 × 10^3^–4.32 × 10^3^ cm^−3^ for total particle and UFP number concentrations, respectively (Figure 6). The highest daily average total particle and UFP number concentrations were observed on 14 February 2014, and they were 1.08 × 10^4^ and 5.50 × 10^3^ cm^−3^, respectively, corresponding to the firework display. The lowest daily average total particle and UFP number concentrations were observed on 19 February 2014 due to its highest daily average wind speed and they were only 2.84 × 10^3^ and 1.88 × 10^3^ cm^−3^, respectively. The UFP number concentrations at the rural area considered in the current study were remarkably lower than those in urban areas, which have been determined in past studies [29,30,31,32,33,34]. 

In this study, the hourly average ratio of UFP-to-total particle was 55.1% during the whole monitoring period, and the interquartile range was 47.9%–63.4%. The relatively high ratio value of the UFP-to-total particle usually can be observed during noon to afternoon hours, corresponding to photochemical nucleation processes under higher temperature conditions. Otherwise, the relatively low ratio value of the UFP-to-total particle occurred during pre-dawn hours, which was due to the coagulation of aged aerosols. The low ratio value of the UFP-to-Total particle also could be observed after a couple of hours of the agricultural residues burning. Particle coagulation played a crucial role in removing ultrafine particles in the ambient air at high particle number concentrations.

**Figure 7:**
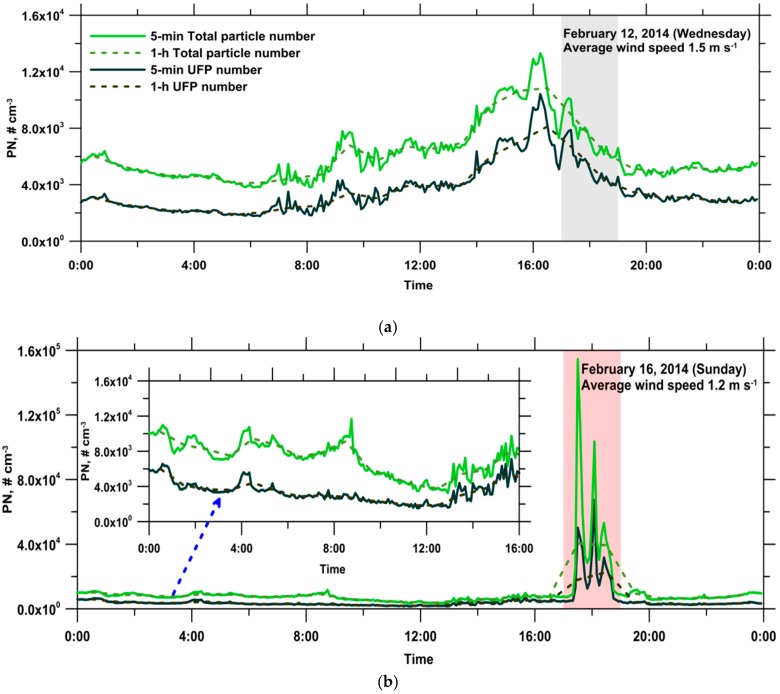
Diurnal variations of 5-min and 1-h total particle and UFP number concentrations at the monitoring site on (**a**) the reference day; and (**b**) the event day.

Figure 7 shows the diurnal variation of the 5-min and 1-h total particle and UFP number concentrations at the monitoring site on the reference and event days. Time variations similar to those of the BC concentrations were observed for the total particle and UFP number concentrations, indicating that the ambient particles could share a common source with BC aerosols in this rural area. The daily average total particle and UFP number concentrations were 6.13 × 10^3^ and 3.61 × 10^3^ cm^−3^ for the reference day, respectively. On the event day, the daily average total particle and UFP number concentrations were 1.01 × 10^4^ and 4.83 × 10^3^ cm^−3^, respectively. On the reference day, the total particle and UFP number concentrations did not vary appreciably between 01:00 and 06:00. The lowest total particle and UFP number concentrations were observed during the interval of 04:00–06:00, and they were 4.13 × 10^3^ and 1.97 × 10^3^ cm^−3^, respectively. Subsequently, the particle number concentrations increased from 07:00 to 16:00. Between 15:00 and 17:00, the highest total particle and UFP number concentrations were 1.07 × 10^4^ and 7.57 × 10^3^ cm^−3^, respectively, and these values were approximately 2.6 and 3.8 times the lowest values, respectively. Thereafter, the particle number concentrations decreased gradually from 16:00 to 00:00, returning to the background level. On the event day, an abrupt increase in the total particle and UFP number concentrations was clearly observed during the corn straw open-field burning episode (17:00–19:00). 

### 3.4. Characteristic of Particle Number Size Distribution during Corn Straw Open-Field Burning 

**Figure 8:**
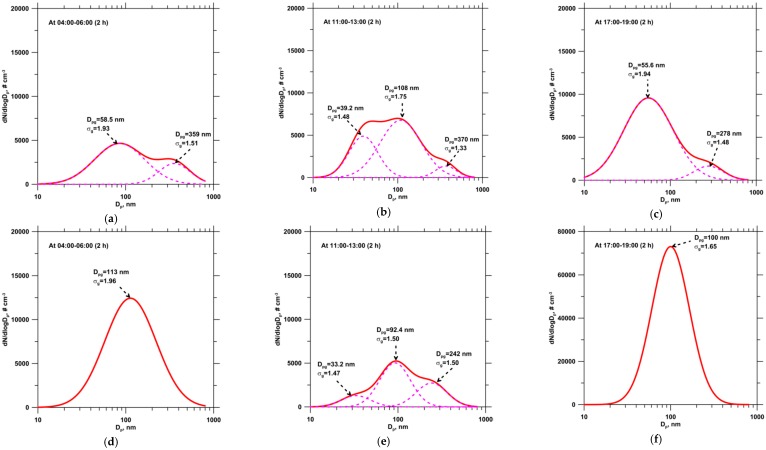
Average particle number size distributions measured on reference day during (**a**) predawn hours; (**b**) midday hours; and (**c**) evening hours; and on event day during (**d**) predawn hours; (**e**) midday hours; and (**f**) evening hours.

Figure 8 shows the average particle number size distributions measured on the reference and event days in three different periods, predawn (04:00–06:00), noon (11:00–13:00), and evening (17:00–19:00) hours. Here, the measured particle number size distributions were determined using DistFit software (Chimera Tech., Forest Lake, MN, USA). The fitting results demonstrated that the number size distribution patterns at the monitoring site could be accurately simulated using log-normal models. According to the measurement results, the particle number size distribution patterns for different periods were considerably different. On the reference day, the particle number size distribution during the predawn hours exhibited two modes: the Aitken mode, which was the major mode, and accumulation mode, which was the minor mode, corresponded to particle diameters of 59 and 359 nm, respectively. This two-mode pattern could usually be observed in the general ambient atmosphere. Additionally, a new mode was observed at 39 nm during midday hours. This new mode of the particle number size distribution was probably associated with photochemical nucleation processes. Similar patterns at midday hours were also observed on other monitoring days. During evening hours, the particle number size again showed a two-mode distribution, with the major mode at 56 nm and the minor mode at 278 nm. During predawn hours on the event day, the particle number size distribution exhibited a single-mode pattern, and the dominant mode was at 113 nm. This single mode of the particle number size distribution likely corresponded to an increase in household activities and emissions from residential wood burning. The pattern of the particle number size distribution during midday hours on the event day was similar to those on the reference day. Nevertheless, the nucleation mode of the particle number size distribution was not remarkable comparing to that on the reference day due to the different intensity of photochemical reaction. During corn straw open-field burning, the dominant mode of the particle number size distribution was at 100 nm.

Reid et al. [54] noted that the count median diameter for fresh biomass smoke was in the 100–160 nm range with a 1.5–1.9 geometric standard deviation. However, studies on particle number size distributions of smoke emissions from the burning of agricultural residues are scant. By simulating agricultural fires, Hays et al. [13] illustrated that particle emissions from wheat straw burning had a unimodal size distribution in the accumulation mode and that the mode peaked at approximately 120 nm. By using a combustion stove in a laboratory, Zhang et al. [5] estimated that particle number size distributions for rice, wheat, and corn straw burning aerosols had a single accumulation mode, with peaks at 100, 150, and 150 nm, respectively. The different dominant modes of corn straw burning between this current study and previous laboratory studies could be caused by their different combustion conditions. Janhäll et al. [55] suggested that the particle size distribution of biomass smoke was dependent on plume age, fuel type, and combustion efficiency. Despite the different sizes of the dominant mode compared with those of previous studies, the measurement results in the current study showed a single-mode pattern, similar to that observed in previous studies, for the particle number size distribution associated with agricultural residue burning.

### 3.5. Short-Term Effect on the Air Quality during the Episode Period 

**Figure 9:**
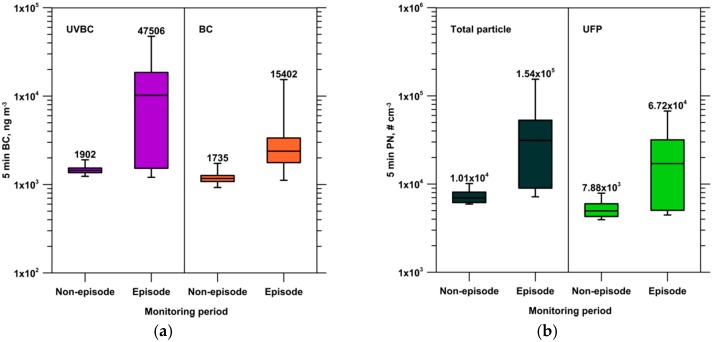
Five-minute concentration levels of (**a**) UVBC and BC; and (**b**) total particle and UFP during the non-episode and episode periods. The box and whisker plot show the lowest, lower quartile, median, upper quartile, and maximum values.

Figure 9 shows the 5-min concentration levels of UVBC, BC, total particle, and UFP during the non-episode and episode periods. During the episode, the 5-min UVBC mass concentration can reach 47,506 ng·m^−3^, and, remarkably, it is approximately 25.0 times the value in the non-episode period. The 5-min BC mass concentration during the episode could increase to 15,402 ng·m^−3^, which was 8.9 times the value in the non-episode period. The hourly UVBC and BC mass concentrations during the episode increased to 11,932 and 3775 ng·m^−3^, respectively, which were 8.3 and 3.2 times the values in the non-episode period, respectively. During the episode, the 5-min total particle and UFP number concentrations could reach 1.54 × 10^5^ and 6.72 × 10^4^ cm^−3^, respectively, which were 15.2 and 8.5 times the values in the non-episode period, respectively. These particle number concentrations during the episode were comparable with those at a busy traffic site and could severely affect human health. Additionally, the hourly total particle and UFP number concentrations during the episode increased to 3.99 × 10^4^ and 2.03 × 10^4^ cm^−3^, respectively, which were 5.3 and 3.8 times the values in the non-episode period, respectively.

Table 2 shows the hourly average particle number concentrations for the episode and non-episode periods for seven selected particle size ranges: 18–32, 32–56, 56–100, 100–180, 180–320, 320–560 nm, and other sizes (comprising the ranges 13–18 and 560–750 nm). Particles in the size range of 100–180 nm (38.1%) and 56–100 nm (37.6%) were dominant during the episode. By contrast, particles in the size range of 32–56 nm (28.7%) and 56–100 nm (28.3%) dominated during the non-episode period. The measurement results showed that a large amount of aerosol was produced during the episode period, degrading the air quality in the surrounding areas. The BC mass and particle number concentrations at the ground level near the burning site were far higher than the values measured at the monitoring site, which was located on the rooftop of a 15-m-high building. Therefore, the risk posed by the high short-term BC mass and particle number concentrations to public health should be addressed. 

### 3.6. Relationship between BC and Size-Resolved Particle Number Concentrations during the Non-Episode and Episode Periods 

Little information is available on the correlation between BC mass and size-resolved particle number concentrations for agricultural residue burning. Table 3 presents the correlations between the BC mass and particle number concentrations for seven size ranges for the episode and non-episode periods. According to the measurement results, the 5-min BC mass concentrations were significantly and positively correlated with the 5-min particle number concentrations for the size range of 100–180 nm (*R_Pearson_* = 0.910), and less significant positive correlations were also observed for the size ranges 180–320 nm (*R_Pearson_* = 0.845) and 56–100 nm (*R_Pearson_* = 0.827) during the episode. The low correlation between the 5-min BC mass and particle number concentrations for the size range of 18–32 nm could be attributed to the highly variable particle number concentrations during the monitoring period. Particles in the size range below 32 nm released during agricultural residue burning could grow rapidly through gas-to-particle reactions or coagulation in the atmosphere. The measurement results indicate that most of the BC in fresh aerosols during the episode could range from 56 to 320 nm in size. Additionally, the correlation between the 5-min BC mass and particle number concentrations during the non-episode period was weaker than that during the episode, and they were correlated for the particle size ranges of 180–320 (*R_Pearson_* = 0.566) and 320–560 nm (*R_Pearson_* = 0.597). The measurement results implied that the aged BC aerosols in the atmosphere contained larger particles compared with the fresh BC aerosols formed by coagulation, and the aged BC aerosols typically reside in the accumulation mode size range. 

## 4. Conclusions

In this study, corn straw open-field burning was clearly observed upwind of the monitoring location between 17:00 and 19:00 on 16 February 2014. The 5-min BC concentration during the corn straw burning period increased to 15,402 ng·m^−3^, which was 8.9 times the value during the same period on the reference day. According to the field measurement results, the hourly absorption Ångström exponent value was 2.0 during the episode. This value implies that the source of BC aerosols was related to agricultural residue burning during the episode. During the episode, the 5-min total particle and UFP number concentrations reached 1.54 × 10^5^ cm^−3^ and 6.72 × 10^4^ cm^−3^, respectively. These particle number concentrations were comparable with those at a busy traffic site and could severely affect human health. Furthermore, the particle number concentrations during the episode were dominated by particles in the size range of 56–180 nm. The measurement results showed a single-mode pattern for the particle number size distribution, and the dominant mode was at 100 nm. They also suggest that most of the BC mass concentrations in fresh aerosols during the episode were significantly and positively correlated with particle number concentrations in the size range of 56–320 nm, implying that BC in the fresh corn straw burning aerosols could be in this size range. According to toxicological studies, these BC aerosols in the ultrafine size range may be more harmful to health than large particles are. Therefore, future studies should address the risk to human health from exposure to high BC mass and UFP number concentrations during a short period. 

## Figures and Tables

**Table 1:** Meteorological data for the corn straw open-field burning period and for the same period on the reference day.

Monitoring Period	Wind Speed m·s^−1^	Wind Direction	Temperature °C	Relative Humidity %
Non-episode period ^a^	1.6	NNW-NNE	16.2	68.4
Episode period ^b^	1.1	NW-N	19.7	71.8

^a^ Data monitored at 17:00–19:00 on reference day; ^b^ data monitored at 17:00–19:00 on event day.

**Table 2:** Particle number concentrations in seven selected fractions during the corn straw open-field burning period and the same period on the reference day.

Monitoring Period	18–32 nm	32–56 nm	56–100 nm	100–180 nm	180–320 nm	320–560 nm	Other Size ^c^
Particle number, cm^−3^							
Non-episode period ^a^	1.12 × 10^3^	2.15 × 10^3^	2.12 × 10^3^	1.05 × 10^3^	5.83 × 10^2^	2.68 × 10^2^	1.85 × 10^2^
	(15.0%)	(28.7%)	(28.3%)	(14.1%)	(7.8%)	(3.6%)	(2.5%)
Episode period ^b^	3.58 × 10^2^	4.47 × 10^3^	1.50 × 10^4^	1.52 × 10^4^	3.74 × 10^3^	5.07 × 10^2^	1.36 × 10^2^
	(2.1%)	(11.2%)	(37.6%)	(38.1%)	(9.4%)	(1.3%)	(0.3%)

^a^ Data monitored at 17:00–19:00 on the reference day; ^b^ data monitored at 17:00–19:00 on the event day; ^c^ other size: 13–18 nm and 560–750 nm.

**Table 3:** Pearson correlation coefficients between the BC mass and particle number concentrations for seven selected size ranges during the corn straw open-field burning period and the same period on the reference day.

Monitoring Period	18–32 nm	32–56 nm	56–100 nm	100–180 nm	180–320 nm	320–560 nm	Other Size ^c^
Non-episode period ^a^	0.209	0.090	0.041	0.195	0.566	0.597	0.239
Episode period ^b^	0.107	0.518	0.827	0.910	0.845	0.794	0.643

^a^ Data monitored at 17:00–19:00 on the reference day; ^b^ data monitored at 17:00–19:00 on the event day; ^c^ other size: 13–18 nm and 560–750 nm.
